# Prospective Health Impacts of a Universal Basic Income: Evidence from Community Engagement in South Tyneside, United Kingdom

**DOI:** 10.1177/27551938241265928

**Published:** 2024-08-01

**Authors:** Neil Howard, Grace Gregory, Elliott A. Johnson, Cleo Goodman, Jonathan Coates, Kate E. Pickett, Matthew T. Johnson

**Affiliations:** 1 Social & Policy Sciences, 1555 University of Bath, UK; 2 Social Work, Education and Community Wellbeing, 5995 Northumbria University, UK; 3 Basic Income Conversation, UK; 4 St Anthony's Health Centre and Department of Anthropology, 3057 Durham University, UK; 5 Department of Health Sciences, 8748 University of York, UK

**Keywords:** universal basic income, health impacts, community development, pilots, Levelling Up

## Abstract

Studies have suggested that universal basic income (UBI) has the capacity to have substantial health benefits across the population at national level. Multiple impact pathways have recently been theorized and there are calls for trials to explore these pathways empirically. However, very limited research has taken place at local levels to explore potential context-specific effects, or how these effects could play out in economic, social, and behavioral changes. In order to examine these effects and to think through potential issues and unintended consequences, we brought together citizen engagement groups in Jarrow, South Tyneside, in the northeast of England to explore local people's expectations and positions on the development of UBI policies and pilots prior to their implementation. We found that people's expectations regarding the potential beneficial health impacts of UBI on their communities mapped strongly onto academically theorized impact pathways. They also extended understanding of these pathways in meaningful ways. Our findings add to the literature about UBI and health and provide important insights for the future development of empirical, health focused, UBI research.

A growing body of evidence suggests that universal basic income (UBI), a system of regular, secure cash payments to all citizens or permanent residents, may have the capacity to generate substantial health benefits across the population at national level.^1^ These studies draw on data from past trials of UBI-like interventions, from UBI-style policies such as the Alaska Dividend Fund, from cash transfer studies, from wider work on the economic determinants of health (such as that featured in *The Lancet Public Health*),^2^ and from microsimulation based on observational data on income increases.^3,4^ Although these studies warn against the difficulty of extrapolating any solid conclusions about UBI from non-UBI interventions, they nevertheless point squarely in two broadly intuitive directions: (*a*) many negative health outcomes are causally related to poverty, precarity, and inequality; ergo (*b*) through tackling poverty, precarity, and inequality, UBI could represent an impactful upstream public health intervention that reduces negative health outcomes, especially among the poorest.

This argument has most recently and fully been theorized by Johnson and colleagues in a series of publications outlining “The Health Case for Basic Income”.^5,6^ The visual model of this argument can be seen in Figure 1 (adapted from Johnson and colleagues).^5^ It features three separate but interrelated biopsychosocial pathways to impact on health. The first relates to poverty reduction and the impact that having greater resources has on satisfaction of needs. The second focuses on stress and the positive health benefits of its reduction through the provision of predictable and secure income that cannot be removed. The third relates to health behaviors, and the role that unconditional, regular cash, as well as mitigation of harmful psychological effects of inequality, plays in enhancing long-term thinking and investment in health-promoting behavior and mitigation of short-term coping strategies, such as smoking.

**Figure 1.**
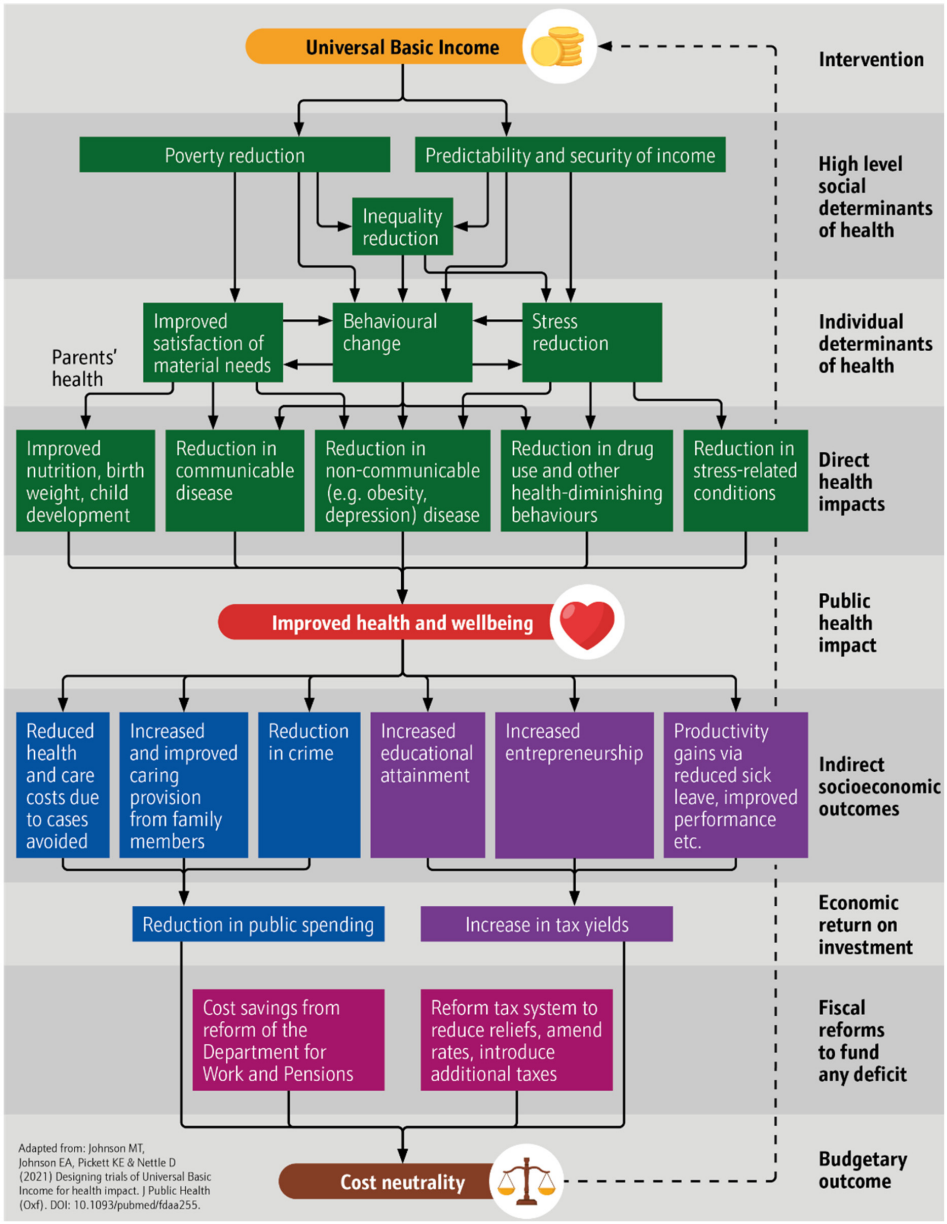
Original UBI model of health impact.

However, to date there has been very limited research at local levels to explore people's perspectives on potential local effects of a UBI, or how those effects could play out in economic, social, and behavioral changes. In order to examine perspectives and to think through potential issues and unintended consequences regarding piloting UBI, we brought together citizen engagement groups in Jarrow, South Tyneside, in the northeast of England to explore local people's expectations and positions on the development of UBI policies and pilots towards their implementation. We found that people's expectations of the potential beneficial health impacts of UBI on their communities mapped strongly onto academically theorized impact pathways. They also extended these pathways in meaningful ways. Our findings thus add to the literature on UBI and health and provide important insights for the future development of empirical, health focused, UBI research.

## Methods

The authors of this article are part of an initiative to develop a costed proposal for UBI micro-pilots in England.^7^ This initiative unites partners from several universities and civil society institutions. Initial expressions of interest in taking part in a micro-pilot from a local community organization, Big Local Central Jarrow, led the project team to explore the community of Jarrow, South Tyneside, in the northeast of England as a potential location for the proposed pilot. The town, which had a population of 29,467 in 2021 and is part of the Tyneside conurbation,^8^ which had a population estimate in 2013 of 832,469 and is served by the Tyne and Wear Metro underground and overground train service.^9^ Jarrow is representative of the kind of low-income community likely to be most affected by the introduction of a UBI, an archetypal target for the UK government's “Levelling Up” policy.^10^ The South Tyneside local authority was the 11th most deprived in England in 2019 for health and 22nd based on the overall Index of Multiple Deprivation (IMD) (MHCLG). Modeling suggests that greater benefits from UBI would result to those lower down the socioeconomic ladder.^11^

The research team held two two-hour workshops at Big Local Central Jarrow with the same 20 participants in each. Participants were recruited by Big Local community workers using social media and word of mouth. Recruitment purposefully aimed to ensure coverage of each of the four main adult generational groups—Baby Boomers (born 1946–1964), Generation X (born 1965–1980), Generation Y/Millennials (born 1981–1996), and Generation Z (born 1997–2012)—to enable workshop findings to reflect concerns across the life course. Care was also taken to ensure gender balance and diversity in terms of occupation and socioeconomic status. All participants were remunerated for their time at university research assistance rates in accordance with NIHR standards to address ethical concerns about exploitation of research participants. In each workshop, participants were split into three roughly equally sized groups, Baby Boomers, Generation Z, and Generations X and Y (the latter two generational groups were combined because participants from each were too few in number). All groups were accompanied by a facilitator to guide conversation and prompt discussion of hopes, desires, and concerns related to UBI and UBI piloting. Each group also had a notetaker who recorded the conversation and noted key observations.

In the first workshop, “Understanding the Feasibility and Desirability of a Universal Basic Income Pilot,” conversation was framed with the following big-picture questions: “If a pilot were to happen here, what should it look like? What would your hopes be for this pilot? What of your worries? How could it be designed to deal with those worries?”.^12^ The second workshop (“What Impact Would a National UBI Have Here?”) built on the findings of the first but sought to explore in greater depth people's perspectives on UBI as a potential social policy. This session sought to examine the prospective positive and negative impacts a UBI could have on a community like Jarrow, with a focus on work, precarity, poverty, well-being, and, of course, health. Participants were presented with three UBI schemes of different payment size, ranging per week from £41 per child and £63 per adult under age 65 to £63 per child; £145 per adult under age 65 to £95 per child and £230 per adult as described by Reed and colleagues.^11^ Participants were informed that the intention is to move incrementally through the schemes over the course of time. Necessarily, there was overlap in terms of content and the results of both workshops inform this article. A sister paper (redacted) focuses on local implementation concerns while this article covers health.

After the workshops, transcripts and handwritten notes were thematically analyzed by the research team to draw out key trends in participant responses. Northumbria University's Ethics Committee approved the study prior to commencement. The following section presents the results of that process.

## Results

Participants across our age cohorts were attracted to the idea of UBI as a social policy and felt confident that it could have significant beneficial health impacts in their community. Rooting their analysis in their lived experience of life in Jarrow, they outlined multiple overlapping pathways through which those impacts could occur. This section will outline the pathways, paying attention to differences and similarities across our age cohorts. The pathways can be summarized as:

1. Improving relationships with work
2. Reducing exposure to stress
3. Enhancing freedom over use of time
4. Increasing healthy behaviors and decreasing unhealthy ones
5. Radiating of individual benefits out to the community

Negative potential pathways were also identified, focusing on:

1. More bingeing due to increased availability of resources
2. Theorized negative economic effects like inflation caused by greater spending
3. Theorized negative social effects due to not needing to work

Significantly, participants across all groups emphasized that the starting point for understanding any impacts in Jarrow must be poverty. “Poverty is everything,” said one woman in the Generation Y cohort, and her sentiment was echoed across all conversations. Given this feeling, each of the impact pathways we discuss below must be understood in relation to *the generalized context of poverty*.

A second, vital factor forming a structural backdrop for understanding the impact pathways that people identified was *the general inefficiency, ineffectiveness, and outright indignity of the current social security system*. This point was emphasized by every age group and in all breakout sessions across the two workshops, bar one with the Baby Boomers. In the words of one man from Generation Z, to the general agreement of his peers, “It's not like they’re helping you. They’re not giving you the help that you need. They’re just on your back, pushing you. . . .”

### Positive Health Pathways Proposed by Participants

Improving Relationships with Work. Freedom from financial pressures that “push” people into inappropriate work was at the heart of participants’ views on how UBI might improve relationships with, and well-being through, economic activity. For example, one of the women in the Generation Y group had worked in factories for her entire life. She said that it was common for women to return to work quickly, even after major surgery, because sick pay was simply insufficient to meet their basic needs. She thought a UBI would reduce the material compulsion beneath such decisions and thus enable people to take time away from work to properly heal when necessary. Others in the same group agreed and added that a UBI could go further, enabling people to refuse difficult, dangerous, or undignified work, and instead choose something better. This sentiment was echoed within the younger Generation Z group, who further underlined the choice that UBI might offer to people to take periods of time out of paid work and re-train for more meaningful employment. Indeed, members of this group gave examples of the type of training they would undertake to allow them to progress their careers, instead of being stuck in unfulfilling jobs.

*Reducing Exposure to Stress*. Intimately related to the effects on work were the hypothesized effects on people's stress. Participants consistently shared the message that being poor is stressful; it involves constant worry over how to meet basic needs and it involves the frequent, exhausting juggling of work. One of our Generation Y participants, for example, had to manage five different, zero-hours contracts just to get by, with no regular certainty either about the work on offer in any given week or the income that it would provide. This participant saw UBI as a way out of such precarity, providing a stress-reducing material floor on which he could stand, which would free him from the exhausting cycle of irregular, short-term, often poor-quality employment. His opinion was shared by a young woman from Generation Z:

I think it's the stress of not knowing, like not being able to put food on the table, like you’ll have money to like to fall back on. That stress would be gone pretty much instantly with a UBI.

The current structure of the social security system is also significant here. As one man from Generation Y put it:

They’re not giving you the help that you need. They’re just on your back, pushing you towards menial jobs, low paid crap, shitty work that this government obviously would like to see everybody in—low paid, underpaid work, where you can’t actually buy food, so you go into food banks as well. Hopefully UBI would get rid of that.

Across our groups, and particularly among those of working age, there was general dislike and distrust of the social security system, which was viewed as insensitive, punitive, and abusive, frequently either forcing people into pointless, “make-work” activities that benefitted almost no-one, or treating those unable to work with distrust. This created a negative cycle of ill-being as it massively increased people's stress. One disabled man from the Generation X group shared the following anecdote on this point:

To me, a basic income would feel far more dignified. I mean, we’ve been through some awful things. Still going through them . . . I get people every four years coming to my house asking whether I’m still as blind as I was four years previously . . . and a lot of our income . . . hinges on that. So that happens every four years, which is stressful . . . because straightaway . . . you know they’re coming from a point of view of “we don’t trust you.”

Unsurprisingly, therefore, replacing dehumanizing welfare structures with an unconditional alternative was viewed across Generations X and Y, and to a similar extent among Generation Z, as a surefire way to reduce people's stress and improve their mental health.

*Enhancing Freedom Over Use of Time*. Participants suggested that UBI could have beneficial impacts on their community through its ability to give individuals greater freedom over how they use their time. As with the above points, at root here is the idea that by providing a stable, secure, and sufficient material floor, UBI could remove the survival-related compulsion that is one of the defining characteristics of life in poverty. When extending their imagination to what might be different in this counterfactual world of sufficiency, our participants consistently pointed towards likely health-benefitting changes in time use. One woman in our Generation Y cohort said simply, “With a UBI, you could take care of yourself as well. Take care of your mental well-being, just go for a walk or chat to your kids or your family.” Another added:

Maybe the reason why we find it difficult to eat healthy or to exercise or to find the things that we enjoy doing is partly because our brains are changed because of the stress that we’re under . . . so I wonder whether actually just having a different system where we have that money would mean we were under less stress and have more chance. *Female Participant, Generation Y*

*Increasing Healthy Behaviors and Decreasing Unhealthy Ones*. Changes in individual time use, improved financial security, and reduced stress were linked by participants with anticipated changes in behavior, with participants across age cohorts broadly coalescing around the idea that UBI would increase healthy behaviors and decrease unhealthy ones. Multiple participants, for example, suggested that people would be able to eat more healthily because they would finally be able to afford healthy food. Others suggested that people would exercise more or, in the case of one of the Baby Boomers, “go on holiday.” Participants further suggested that people would leave stressful jobs, be able to invest in house repairs and, if necessary, end toxic relationships. Critically, participants across a range of age groups agreed that alcohol and drug use would likely decrease, since people would have less need to escape from the stress, suffering, and hardship of daily life. One female community worker from Generation Y spoke to this point:

We run a project for people who use substances, and we support substance misuse workers to understand the background that trauma has for people who end up using substances, and I think it is really, really profound, how much of a role poverty plays—like I can’t, I don’t think I can even explain how important it really is. A UBI would definitely help with that.

*Radiating of Individual Benefits Out to the Community*. Intriguingly, changes in time use and the shift towards healthy behaviors were consistently framed by our workshop participants in community as well as individual terms. That is, participants suggested that individual changes would be prosocial and thus beneficial for those beyond any individual UBI recipient. For example, parents pointed to the increased time they would spend caring for their children and the well-being benefits that all would enjoy as a result. Others went further, arguing that UBI would liberate people's “contribution energy” and free them and the community as a whole to engage in life-affirming, communal activities. A female community worker in our Generation X group outlined the following such scenario:

I do think that [by] having this extra money there's going to be pros and cons . . . but I think some of the pros we’re talking about relate to poverty. Like, if you’ve got extra money, you maybe wouldn’t have to be working all hours, like carers, having to work all hours under the sun. You could have extra time where you could do community-based things, like . . . community allotments, where you grow your own food amongst the community, share amongst the community, educate each other about things like “you don’t have to be taking drugs, you can take your mind off things in other ways.”

Other members of this group concurred, and the conversation ranged for ten minutes over how community life might flourish again once the “better angels of our human nature,” as one man put it, had time and space to take flight.

### Negative Health Pathways Proposed by Participants

Before we conclude this section, it is important to note that our participants were not all convinced of the unambiguous health benefits of a UBI. Baby Boomers in particular worried that alcohol abuse would increase as people “pissed away” the extra resources they had. Younger participants too thought there was a danger of a spike in drug use at the start of a UBI program, although they expected this to taper off quickly as the wider, beneficial effects of increased security kicked in. Some older and middle-aged participants, but also someone involved in a small business within the younger group, worried about negative feedback effects like inflation caused by UBI increasing uncertainty and stress, while Baby Boomers worried that people might leave their jobs and “get into the habit of not working,” which could be detrimental personally and socially.

## Discussion

The emerging literature on UBI and health features, in part, what can be characterized as a counterfactual argument based on the well-established relationship between poverty and various dimensions of ill-being and ill-health. This argument takes the form of, “If ill-being and ill-health are in multiple ways causally dependent on poverty, then addressing poverty could provide multiple pathways towards improving health and well-being.”

Our engagement with members of the type of community that this model predicts will most likely benefit from a UBI provides overwhelming support for the impact pathways that it theorizes. Our participants clearly believe that the material security provided by a UBI would improve their physical and mental well-being, reduce their stress, and limit the indignities they presently face at the hands of the U.K. social security system. It would make eating good food easier, facilitate more time with loved ones, and liberate people to make different—and potentially more beneficial—choices over their present and their future. This impact on decision making, it was suggested, would translate into increased health-promoting behavior and a reduction in activities like drug-taking, which participants reported to be rife in Jarrow as a way for people to escape their misery and stress. Evidence examined in Gibson, Hearty, and Craig^1^ suggests that bingeing is associated with large, irregular payments and that regular payments of modest amounts are unlikely to lead to long-term problems. To this extent, the findings from our qualitative community engagement strongly echo this established model.

However, we believe that they also point in the direction of a significant gap in that model, regarding the *relational impacts* of UBI and the beneficial feedback that these impacts could have on individual and collective health and well-being. As a female community worker from Generation X put it:

I think the universal basic income, it's going to free up people's time a little bit, to do other things to help each other. Like I said before, if you’re not working 16 h a day and sleeping for four hours, you’ve got that extra money where you can contribute to helping be a community again and caring about each other again, and it doesn’t just necessarily have to just be just Jarrow, care about society as a whole.

**Figure 2.**
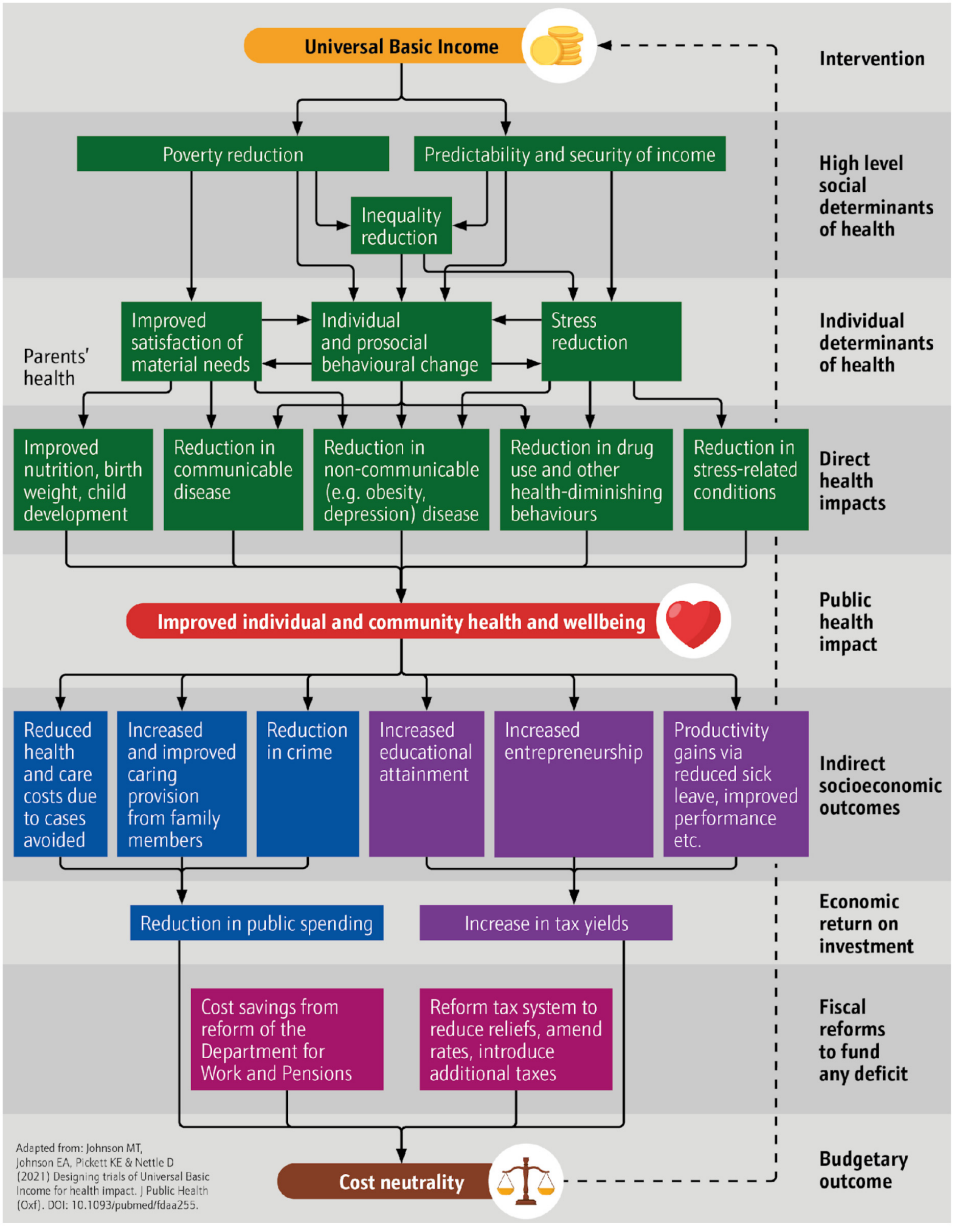
Updated UBI model of health impact.

While plans elsewhere, including Finland, found little negative impact on labor market participation,^13^ one strand of the wider UBI literature has long theorized that by freeing people from economic compulsion, basic income would allow them to invest their time prosocially in cooperative, collaborative, and non-market-based community activities.^14,15^ This literature implicitly affirms a view of human nature as prosocial, wired for connection, and prone to compassion. It therefore argues that when they have the chance to do so, people will gravitate towards the kinds of collective activities that have been well documented to foster “relational well-being”.^16,17^ Importantly, UBI trials have produced empirical evidence of this. In Namibia, for example, studies have documented that participants in the country's UBI pilot experienced an increase in community spirit “leading to higher levels of community activity, mutual social ties, and participatory community engagement and interaction”.^15^^, p. 81;^ ^18, 19^ In an age of alienation and dislocation, what has been described as “an epidemic of loneliness”^20^ has emerged that has been found to have annual well-being, health, and productivity impacts of at least £9,976 per person experiencing severe loneliness^21^ and £2.5 billion for employers due to loneliness overall.^22^ With this study identifying potential impact in this area, we suggest that the health case for basic income may need to be refined to take account of prosocial changes and their health and well-being impacts. We have updated our model of impact in Figure 2 to revise “Behavioral change” to “Individual and prosocial behavior change” as an individual determinant of health and “Improved health and well-being” to “Improved individual and community health and well-being” as the public health impact. These significant conceptual revisions stem directly from community participation.

A significant strength of the study is that it was undertaken in a town, a geographical unit within England and Wales defined by the U.K. Office for National Statistics as having a population between 5,000 and 225,000 residents, with a medium-sized town like Jarrow having a population between 20,000–75,000.^8^ Such towns are often overlooked in policy analysis in favor of cities at one end of the scale and rural areas at the other. Addressing this imbalance is essential as around one third of England and Wales’ population,^23^ some 21.6 million people, lived in small or medium towns in 2019.^8^ In addition, Jarrow is typical of “red wall,” traditionally Labour Party-supporting constituencies that have, in recent years, become important targets by both main U.K. political parties after significant swings to the Conservatives in 2019.^24^

In terms of limitations, necessarily, our study is qualitative and small-scale, so definitive conclusions cannot be drawn. It is plausible, for instance, that other deprived communities would have different views on the potential impacts of a UBI, or that they would view it as likely to be damaging. Further research is therefore imperative.

Perhaps more importantly, however, a broader tranche of research is required in order to understand the impacts of UBI as a redistributive measure on wealthier, more secure communities. A body of evidence indicates that highly unequal and insecure societies produce outcomes even for wealthier members of society that are lower than those of more equal and secure societies.^25^ This may be due to reduction in trust, cooperation, and prosociality that affects all members of society. There is need to examine the prospective impacts of UBI on groups that might be liable to pay more in tax to fund such plans in order to understand the prospective trade-offs envisioned. This research could take place in relatively proximate communities, such as Jesmond, in Newcastle upon Tyne.

## Conclusion

This article builds the evidence base supporting existing UBI models of health impact and provides indications for further development, with an emphasis on relational community benefits radiating out from individual impacts. It also provides further empirical evidence on the benefits of ethical coproduction with communities affected by policy, in particular upstream economic interventions. This helps to clarify important pathways for impact among (public) health practitioners too, suggesting that a shift to economic interventions may be necessary to address a range of population health issues, and/or that consideration should be given to identifying whether and how alternative social, medical, and behavioral interventions are able to impact those pathways.
